# Managing Substance Use Disorder through a Walking/Running Training Program

**DOI:** 10.1177/1178221820936681

**Published:** 2020-07-06

**Authors:** Chia-Liang Dai, Ching-Chen Chen, George B Richardson, Howard R. D. Gordon

**Affiliations:** 1 Department of Teaching and Learning, University of Nevada, Las Vegas, USA; 2 Department of Counselor Education, School Psychology, and Human Services, University of Nevada, Las Vegas, USA; 3 School of Human Services, University of Cincinnati, USA

**Keywords:** supervised exercise program, boot camp workouts, substance use disorder, relapse prevention

## Abstract

While emerging studies have demonstrated the benefit of exercise in Substance Use Disorder (SUD) recovery outcomes, lack of motivation to engage in exercise has been indicated as one of many perceived barriers that contribute to low recruitment and adherence rates in SUD treatment. The current study aimed to explore participants’ perceptions of attending a supervised exercise program (boot camp workouts, walking/running practice, and a race event) while in treatment for SUD. A total of 109 participants were recruited to a 14-week exercise training program and 61 chose to participate in, and completed, a race at the close of the program. Interviews were conducted during weeks 6 through 14 and data were examined using Thematic Analysis. Three main themes were identified: (1) pushing forward recovery through running, (2) gaining a sense of achievement by crossing the finish line, and (3) building a sense of belonging in the program. Implications for SUD recovery programs are discussed.

Substance Use Disorder (SUD) affects an estimated 19.7 million people in the United States in 2017^1^ and relapse prevention is a major challenge in healthcare.^2^ SUD patients have an increased prevalence of many severe health consequences including cardiovascular disease, liver damage, arthritis, respiratory deficits, cancers, HIV/AIDS, and mental illness as well as associated injuries, broken relationships, and loss of employment.^3,4^ In 2017, the life expectancy of the United States dropped for the first time in decades; drug (eg, overdose) and alcohol (eg, motor-vehicle injury or violence) related deaths are two major contributing factors.^5^ These costs highlight the importance of substance use prevention and the receipt of treatment services for people experiencing SUDs.

SUD recovery has been defined as a change process of either moving toward continued drug use or abstention from use.^6^ Borkman, Stunz, and Kaskutas defined recovery as a way of being, or state of growth and learning involving internal values and self-awareness.^7^ Similarity, Anthony articulated that “recovery is a deeply personal, unique process of changing one’s attitudes, values, feelings, goals, skills, and/or roles.”^8^ The definition of recovery acknowledged by the Betty Ford Institute Consensus Panel is “a voluntarily maintained lifestyle characterized by sobriety, personal health, and citizenship.”^7^ (p. 222) It seems most recovery definitions suggest “it is a way of living a satisfying, hopeful, and contributing life even with limitations caused by illness.”^9^ (p. 15) The recovery process can be difficult and among those who were diagnosed with SUDs and received treatment, at least 60% experienced relapse within one year.^10^ To facilitate SUD recovery, if the individual demonstrates effective coping strategies in response to the urge and self-efficacy to deal with the situation, they are more likely to change behaviors even when faced with obstacles.^11^ If they feel that they can control their health behavior, they might be motivated to act, or to persist through challenges and experience progress in the recovery process.

A lifestyle-based adjunct to traditional treatment for people experiencing SUDs is involvement in physical activity. Health benefits of regular physical activity is evident, including reduced risk of various chronic diseases, improved concentration, elevated mood, and better sleep quality.^12^ Physical activity is also low cost and has been linked to reduced depression and improved tolerance of stress that may facilitate the recovery from SUD.^13^ Muller and Clausen implemented a 10-week group exercise (walking/running, ball games and strength-training sessions) adjunctive program for SUD patients at a residential treatment center. Results suggested that participants who received the intervention experienced improvement in the physical health (eg, improved fitness), psychological health (eg, reduced anxiety), and domains of quality of life (eg, felt more energetic, better sleep quality) relative to controls.^14^ Exercise influences many of the same signal molecules and neuroanatomical structures that mediate the effects of substance use, consistent with the theory that it can serve as a healthy, non-drug source of reinforcement to decrease substance use.^15^ The effects of exercise on health related outcomes among clients diagnosed SUDs have supported that exercise is a feasible and safe adjunct treatment for SUDs with benefits to physical, psychological, behavioral, neurological, and overall personal health.^13,16–18^

However, Linke and Ussher reported that recruitment of SUD patients to an exercise program is challenging.^13^ In addition, low adherence to exercise programs might undermine their effectiveness.^16^ Other most commonly reported barriers to engaging in exercise programs among people with SUDs included cost, transportation, and equipment.^19^ Although many residential SUD recovery centers are equipped with fitness facilities, the use rate is low. Flemmen and Wang indicated that SUD patients have impaired endurance and muscular strength compared with an age and sex matched reference group, and suffer higher risk of developing other chronic illness and early death.^20^ Findings of another study demonstrated that participants in SUD treatment exhibit low rates of physical activity and low motivation to engage in physical activity; however, participants indicated a tendency to attend if an exercise program was offered during their substance abuse treatment in the recovery facility.^21^ These findings suggested the importance of increasing motivation to engage in physical activity for people in recovery, especially by providing guided and scheduled exercise programs in hope of enhancing participants’ engagement in physical activity.

In view of the complexity of SUD and recovery, it might be crucial to provide activities that can serve as positive coping responses to substance use, and coping strategies that clients can sustain in their life to help manage their SUDs. There is limited research that has explored participants’ perceptions in exercise programs during their recovery. In this study, we implemented a supervised group exercise program, which was composed of boot camp workouts, walking/running practices and a race event, in a treatment facility for SUD. We investigated the perceptions of people experiencing SUDs in a residential recovery center during the 14-week supervised group exercise program.

## Method

This qualitative study investigated SUD patients’ perceptions of participation in a supervised walking/running exercise program. Thematic Analysis was utilized for this study to acquire knowledge about participants’ experiences.^22^

### Participants

Participants (*n* = 109) were recruited from a treatment facility for recovery in a mid-sized city in the Midwest, USA. Inclusion criteria included: resided in the recovery center, older than 18 years old, and provided consent to participate in the walking/running training program. The recovery center serves populations who are economically disadvantaged, low-income, unemployed, underemployed, and dislocated. There were 54% females, and 46% males. The racial makeup of these participants was 63% Caucasian, 28% African American, 3% Hispanic, and the remaining 6% of participants were marked unknown or other. Participants’ age group was made up of 21% 20 to 29 years, 28% 30 to 39 years, 15% 40 to 49 years, 25% 50 to 59 years, and 11% 60 to 69 years. Participants were impacted by frequent and severe use of substances including alcohol, cocaine, heroin etc. The majority (80%) of people who have accessed recovery services at the treatment center had opioid use disorders. Other life experiences reported by participants included homelessness, incarceration, prostitution, and unhealthy domestic issues. A typical stay lasted for 12 months; however, the length of stay was determined by the participant’s individual goals and treatment plan. To provide a safe and healthy living environment that promotes recovery from SUD, the supervised residential recovery center provided participants with treatment components including meal and shelter services, individual and group counseling, educational and vocational training classes, financial counseling, parenting skills training, and aftercare services. Participants were required compliance with all prescribed medication to remain in the program. Random and routine drug screens, alcohol screens with a Breathalyzer unit, and room searches were also conducted.

### Intervention

This 14-week exercise program included four weeks of boot camp (once weekly), nine weeks of walking/running (twice weekly), and the 14th week included the race (see Table 1), which was implemented by a recovery center in partnership with people volunteering to help those recovering from SUD. The goal of the boot camp workouts was to provide body movements that help participants build strength and endurance. Boot camp workouts require little to no specific equipment and create a sense of camaraderie among participants. Walking/running was chosen because there is no specific equipment required except a pair of running shoes. Participants with any medical conditions were directed to the healthcare professional or physician for specific recommendations regarding exercise. Program staff or volunteers also encouraged participants to try walking instead of running if participants indicated any concerns of health risks with running.

**Table 1.** Description of exercise program.

Type	Week	Duration	Event
Bootcamp	Week 1	90 minutes	boot camp1
	Week 2	90 minutes	boot camp2
	Week 3	90 minutes	boot camp3
	Week 4	90 minutes	boot camp4
Walking/ running	Week 5	30 to 60 minutes	Kickoff + first walk/run
	Week 6	30 to 60 minutes	In training
	Week 6	30 to 60 minutes	In training
	Week 7	30 to 60 minutes	In training
	Week 7	30 to 60 minutes	In training
	Week 8	30 to 60 minutes	In training
	Week 8	30 to 60 minutes	In training
	Week 9	30 to 60 minutes	In training
	Week 9	30 to 60 minutes	In training
	Week 10	30 to 60 minutes	In training
	Week 10	60 to 90 minutes	In training
	Week 11	60 to 90 minutes	In training
	Week 11	60 to 90 minutes	In training + informal race event
	Week 12	60 to 90 minutes	In training
	Week 12	60 to 90 minutes	In training + cheer day
	Week 13	60 to 90 minutes	In training
	Week 13	30 to 60 minutes	In training
Race week	Week 14	30 minutes	In training + pre-celebration
	Week 14	N/A	Race day

Boot camp workout was delivered once a week (ie, four sessions) at the facility for the first four weeks of the program. It included intense body movement such as aerobic, strength training, and speed element in each session. Each boot camp workout lasted for one hour and thirty minutes. Walking/running practices lasted from weeks five to fourteen and were held twice weekly outside of the facility. A total of 18 practice sessions were scheduled and implemented. Each walking/running session lasted from the range of 30 to 90 minutes (as the distance of walking/running increased). In the beginning of each training, participants were guided to stretch their body to warm up before walking/running. In the beginning of the program, participants were encouraged to select a type of race they expect to train for as their goal. Volunteers were then partnered with individuals or a group of participants for the rest of the training sessions. During the practice time, the goal was to keep participants engaging in body movement. In each practice, the staff announced the aimed distance of walking/running for each type of race event; participants were encouraged to walk and/or run for the planned distance or time. Participants can walk first to warm up and start to run when they feel comfortable. The staff and volunteers, who were experienced in instructing fitness classes and running regularly, led both boot camp and walking/running sessions.

Several events such as athlete of the week, participation of a local running event, cheer up day, pre-race lunch, and post-race celebration were implemented along with the walking/running practices. To recognize participants’ engagement in the exercise practice, athlete of the week is selected and voted by volunteers or endorsed by their running coach or program director. Joining a local running event while in training offered a tune-up run for the participants. Participants experienced the race atmosphere where other runners and community members greeted them. On the cheer up day, live music, food, and the mascot of a professional sports team were there to cheer participants. Pre-race lunch was scheduled one week prior to the race. Participants and volunteers gathered together to celebrate how far they have trained for the race while in recovery. Finally, after the race day, family, volunteers and community members were all invited to join the post-race celebration.

### Data collection

To get a complete sense of how participants experience the program, interviews were conducted beginning in week six and ending after the race. Forty-nine participants completed interviews. During training weeks six through 13, 24 individual interviews were completed. After the race, an additional 25 participants were interviewed. The interviewer asked the participant an open-ended question: describe your experiences in the exercise program. Interviews were conducted in a quiet space inside or outside of the treatment center. Each interview period ranged from 15 to 30 minutes. Interview videos were then transcribed into texts by the authors.

### Data analysis

The data analysis was conducted based on a six-phase process: familiarization with the data, coding, generating initial themes, reviewing themes, defining and naming themes, and producing the report.^22^ Initially, two researchers read the texts and coded them independently using open coding.^23^ The purpose of this step was to simplify the data and allow for emergent categories to be identified.^24,25^ Any disagreement about coding was discussed until consensus was reached over the course of several face-to-face meetings. Codes were then combined into themes, which involved finding general patterns among specific information.^26,27^

### Trustworthiness of data

Lincoln and Guba argued that trustworthiness of a study involves establishing credibility, transferability, dependability, and confirmability.^28^ To achieve these criteria, the authors reviewed transcripts and interviews several times to gain a deeper understanding of the phenomenon and provoke meaningful themes. The codes and themes were then reviewed by experts in qualitative studies to evaluate the analysis method and provide feedback to ensure its validity. The summary of the results were returned to interviewees, and they were asked to review if authors’ interpretation matched their perceptions and experiences.

### Ethics

The research was approved by the university’s research review board (ID: 20132115). The program manager introduced the researcher to participants in one of the group meetings. The researchers reiterated the research procedure and obtained informed consent from all participants. Each participant voluntarily agreed to be interviewed.

## Results

The purpose of this study was to explore perceptions of participants in a supervised group exercise program for people with SUD. This program was 14 weeks in length and 61 (30 females, 31 males) out of 109 participants ultimately chose to participate in, and completed, the race at the close of the program. After analyzing interview data about participants’ perceptions of participating in the walking/running training program on their recovery, the researchers identified three main themes: (1) pushed forward recovery through running, (2) gained a sense of accomplishment by crossing the finish line, and (3) experienced a sense of belonging in the program (see Table 2). Findings suggested that participation of walking/running seemed to be beneficial for improving recovery experiences in this population.

**Table 2.** Classification of main themes and sub-themes.

Main themes	Sub-themes
Pushing forward recovery	Reduced cravings to drug Improved personal health
Gaining a sense of accomplishment	
Experienced a sense of belonging	Strengthened spirituality Connection with volunteers

### Theme 1: Pushed forward recovery through running

In the program, participants engaged in running and walking instead of drug use as a part of their recovery journey. Participants reported that walking/running practices helped them “tough through” the recovery by reducing cravings to drugs, and improving overall personal health such as weight control, feeling good, and being fit.

#### Reduced cravings to drug (*n* = 16)

Several participants indicated that running served as a positive coping strategy and helped them “tough through” the pain of SUD recovery. In referring to positive coping via running, one participant said,

*“Running is the one thing I look forward to the most every week. I’ve traded my addiction that brought me here for running and healing. However, the most important thing for me is to push through the pain on the inside, and that’s what I’ve dedicated myself to now. Running helps me do that.”*

Most participants expressed how they reduced cravings to drugs via running. For example, one participant stated, “I had experienced the urge to use drugs, but that urge went away while running.” Another participant described how the experience of running helps with recovery, “There is no drugs, no glass of wine that ever makes me feel like I feel every single time that I get out and run with the program.” One participant acknowledged how running has transformed negative coping (eg, addiction and cravings) into a positive coping, “I don’t know what I am doing. My thinking is always negative. When I start to run, I know I can do something.”

#### Improved personal health (*n* = 12)

Participants noted another benefit by involving in running/walking is the improvement of overall personal health such as managing weight, quitting smoking, and eating healthier. One participant stated, “I weighed 350 pounds when I came to the (treatment center). I’m 220 now.” Another participant agreed that, “It (the program) inspires me to quit smoking, eat healthier, and just kind of stay more fit. It is like general better life all over.” Several participants mentioned that the walking/running training program played an important role in helping them during their SUD recovery. As a participant shared that, “I will stay in the treatment longer if I get to continue to run or walk with a walking/running training program.” Running may have facilitated attempts to rebuild their lives during rehab and sustain a positive lifestyle during their recovery. Many participants reported that they hoped to continue this (running/walking) after the completion of the walking/running training program and to participate in other races.

### Theme 2: Gained a sense of accomplishment by crossing the finish line

For many of those participants, achieving a goal (in the race) might be the first success they had experienced in a long time. In this study, all participants who started the race completed the race. In reflecting on the feelings of this experience, many completers (*n* = 13) stated that they gained a sense of achievement and completed something they never imagined when crossing the finish line. One participant in recovery said, “crossing the finish line is the best accomplishment I have ever had.” Another participant noted, after receiving the medal at the race, “this was the first time in my life that I saw an entire process through from start to finish. I couldn’t have done it without this team.”

The majority of participants talked about finishing the race, which is a successful experience that has transformed their recovery journey. The “something completed” experience from the training to crossing the finish line helped participants gain confidence in their recovery. One participant described that, “it is kind of something about I am gonna about to finish the race itself is just like the race of life.” Several participants indicated that they did not know they could run and would not have believed that running could help them overcome addiction. A participant shared that, “when we started training, I thought there was no way I could even walk a 5k. Now I’m running the 10k.” Another participant noted after completion of her first 10k “it felt amazing,” she said. “I felt like ‘wow’, if I can do this, I can do anything.”

Throughout the course of walking/running training, participants appeared to gain confidence over their addictions by learning what it takes to be a success in the walking/running journey-self-discipline and commitment. One participant mentioned, “we have come this far, we have got this,” albeit battling with pain from her injury during the course of training. After completion of the race, one participant noted that,

*“I just lost a job opportunity, but that’s OK because of what I learned about determination, discipline and prayer this year with this running program, I know I’ll be OK. I’ll find a job because I will continue to beat my addiction. I now have a community of support around me.”*

### Theme 3: Experienced a sense of belonging in the program

Participants expressed their participation in the walking/running training program helped them find the belongingness which might have been missed for long in their lives. The sense of belonging was fostered through strengthened spirituality and connection with volunteers in this program.

#### Strengthened spirituality (*n* = 10)

Participants experienced that walking and running practice in the program helped them find inner peace and feel settled down during recovery. One participant noted, “I chased drugs and alcohol because I was empty inside. It wasn’t until I started the program that I found purpose. When I’m running, I get an overwhelming sense of peace.” Another participant described that,

*“in the first couple of days, I really didn’t want to be here (laugh). Yeah, it actually took me two months to settle in. But I had the urge to run. It wasn’t so much to the program. It was me trying to settle down in some place where I haven’t done in a long time.”*

Several participants reflected on how their dedication and faith in God helped them face the pain and fear. One participant was significantly involved in drugs and just recently released from a local prison before participating in the walking/running training program. This participant expressed his desire to build healthy relationships, be free from anxiety, and be able to renew confidence because of his “rededicated to God”. Another participant noted, before his first ever trial of a full walking/running in his life, “God said, ‘you think you can do the half (walking/running), I think you can do the full (walking/running).’ I’m nervous, yes, but I’m not fearful. I know this is going to happen.” Another participant shared that,

*“everything has gotten in a circle. I have got everything I need when I came here. That is family and community. I absolutely love these people that the Lord has placed in my life at this time in this journey. I realized I need these people.”*

#### Connection with volunteers (*n* = 22)

Encouragement and company received from volunteers have motivated participants to stay in the training program. One participant lived under a bridge, never changed his clothes, was shot twice, was hospitalized for nine days by a snakebite, lost a toe to frostbite and had a heart attack. He noted that,

*“today due to the help of my friends in the walking/running training program. Man, look, I got to walk. I don’t know how far I have walked today. But I am blessed to have done it. I have so many friends here. You know, I can’t do it. But so many people here always drive me through that nothing can take me down.”*

The support from volunteers in the walking/running training program is a big part of his hope. Another participant noted that, “I never thought I could do something like this. I feel so good. The volunteers here have given me even more motivation to do what I need to succeed.”

In the first week of boot camp, groups were doing leg stretching exercises. One participant remembered that she said, “I can’t do this.” A volunteer in front of her said, “Yes, you can.” From that moment, they formed a wonderful friendship as they trained together. Another participant shared that, “one year ago today, I was strung out on heroin, was homeless and felt worthless. This program has helped me because now I don’t need a drink or heroin. These volunteers make me feel like I belong.” Another participant was addicted to methamphetamine and felt about giving up before joining the walking/running training program who shared how this program helped him heal, “I feel like my life is coming together. . . . The walking/running training program volunteers have stuck with me every step of the way. They just encourage me to keep going, and I love them like my own family.” Several participants were not feeling well physically, but they battled on because the relationships they built with volunteers motivated them. One participant described that, “my leg is killing me, but I don’t want to stop because I don’t want to let the volunteers down.”

Relationships established between participants and volunteers seems to be the key factor to transforming lives. One participant shared that, “I want to be Athlete of the Week so bad. I know I can do it now. I didn’t think I could at the beginning, but (the volunteer) believes in me. So, I know there must be something good in me!” Another participant described that,

*“For a long time, I saw help as a sign of weakness. To have the volunteers come beside me, cheer me on, and give me encouragement. . .embracing volunteer is a good thing. It doesn’t make me weak; it doesn’t make me seem I am afraid of any matter. Having these people come beside me is definitely a blessing in my life.”*

Most participants in the program expressed how appreciative they were that those volunteers would show up on the practice for them no matter what kind of weather conditions. One participant stated,

“*I came to (the treatment facility) to heal. Heal from poor choices; heal from being rejected by my father when I was a little boy, and the devastation me and my family went through; heal from hating myself and then hating others because of the lies I believed. This (training for the walking/running) is opening my eyes to my life and the words that are on our shirts, ‘Let us run with endurance the race God has set before us.’ I just want to heal and for the pain to go away. Thank you to all the volunteers that care about us to come out here in the cold and do this.*”

Participants perceived that through commitment, discipline, teamwork and determination, they had gained more hope in their race to rebuild their lives.

## Discussion

The purpose of this program was to explore participants’ experiences in the walking/running training program during their recovery from SUD. Of the 109 recruited to the program, 61 started as well as completed the race at the close of the program. Participants reported enhanced recovery experiences by participating in the exercise program during the treatment. Specifically, results indicated that participants experienced running/walking as a helpful coping mechanism during recovery, gained a sense of achievement by crossing the finish line, and developed a sense of belonging in the program. Through involvement in walking or running, the program may cultivate a sense of hope for people recovering from SUD. Overall, the walking and running training program seemed to be a promising adjunct treatment to help participants transform their lifestyles during SUD recovery.

The walking and running training program was designed to facilitate recovery through participant adoption of healthy lifestyle-based coping strategies (eg, physical activity, setting and achieving goals). Results of this study indicated that participants showed positive recovery experience through exercise training. Researchers, for example Weinstock et al. and Stoutenberg et al, have argued the benefits (eg, better self-image, and improved fitness, and reduced stress) of engaging in exercise for clients during their recovery.^13,16,17,29^ This study added to the literature by describing participant experiences of an adjunct exercise program for managing SUDs.

Results of the program indicated the importance of incorporation of alternated behavior (eg, exercise) to replace substance use in the cycle of relapse. Re-exposure to substance use associated cues, such as using substances when facing stressful situations, can cause even individuals with years of recovery to relapse. Perceived stress and feeling loss of control can also lead to relapse. Physical activity has been linked to reduced depression and improved tolerance of stress, which may in turn facilitate recovery from SUD.^17^ Exercise as a stress relief strategy, a healthy coping mechanism, can function as a positive reinforcer that stimulates the brain’s reward pathway and mood-boosting neurochemicals. Exercise can also buffer against substance use by enhancing prefrontal cortex function.^15^ Results of this program are consistent with the findings of past research.^30^ For instance, Muller et al. surveyed 591 patients enrolled in the SUD treatment facility. The study found that most respondents reported poor or very poor quality of life; among women, depression was strongly associated with poor and very poor quality of life; among men, physical inactivity was associated with very poor quality of life, as was reporting eating most meals alone. This study concluded that SUD treatment facilities should provide activities that improve physical health, mental health, and social support in the intervention.^30^ Positive and lasting relationships between participation of regular exercise and various types of health benefits have been well chronicled. Results of the program support previous work suggesting that people with SUDs can receive physical and psychological benefits from group exercise.^14^ It is critical to ensure improved access to such evidence-based adjunct treatment services, and integrated with mainstream healthcare for those at risk for or affected by SUDs.

Attrition rate is a threat to the effectiveness of exercise adjuncts to treatment. High attrition rates usually prohibit participants from obtaining the benefit of regular exercise involvement.^16^ In this walking/running training program, participants selected a goal (eg, 5k to full walking/running) for the race (self-efficacy), could be nominated for the athlete of the week and recognized before each practice for the efforts they made toward reaching their goals (positive reinforcements and observational learning), and teamed up running with volunteers (modeling) throughout the training and during the race.^31^ The dynamic and reciprocal interaction of participants (person), exercise as coping response (behavior), and exercise program context and volunteer support (environment) were utilized in hope to sustain participants’ motivation in the walking and running practices.

This program allows participants to choose the type of race they plan to complete. Then a training plan was designed to facilitate participants to reach their goal. Future programs could include components such as goal setting that allows participants to establish their competence and confidence toward the goal. For example, encouraging participants to walk before running, or inviting participants to run for two blocks instead of two miles in the beginning of the training. The Self-Regulation Model^32^ outlines the power of choice in illness self-management. When the stimuli are presented (eg, urge to use drugs, something happens that provokes negative emotions), the individual recognizes the stimuli and affective responses to the stimuli; then choose the coping responses. The coping responses determine the outcomes. Considering the evidence that physical activity may positively impact the mental and physical wellness among people with substance abuse problems, future studies should continue examining the effect of applying exercise as a coping response in substance use prevention and treatment. Future research could consider examining other forms of low-impact physical activity (eg, yoga or gardening) as an adjunct to SUD treatment.

Recovery capital is comprised of four domains (ie, social, cultural, physical and human) of resources and relationships that could be utilized during recovery.^33^ The findings indicated the importance of positive lifestyles (eg, functional fitness, running, walking), increased self-control (eg, goal setting, sense of accomplishment), and enhanced social capital (eg, social support from volunteers) during recovery. Establishing recovery capital should be a focus during treatment, in hopes that clients adopt those resources or healthy lifestyles while returning to society.^34^ This also indicated adaptive continuing care for people who are recovering from SUD is necessary.^35^ If participants can stay in the healthy environment provided by treatment facility and continue receiving appropriate treatment services, they might be more likely to experience positive outcomes.^2^

The cost expenses of substance use on the healthcare and crime-related costs are far above the expenditure on prevention.^36^ Early substance use is a primary risk factor for SUD, future programs might consider including component of exercise in the prevention of substance use initiation and examine the impact of exercise-based program on broader populations. Further research is required to strengthen these conclusions and to inform policy and healthcare systems.

### Implications

Health professionals can encourage clients to engage in any physical activities that clients might be interested in, or search physical activity based program or events at local area, in hopes of enabling clients to experience the benefits (eg, acquiring positive coping, gaining confidence, and establishing positive bonding relationships) of exercise during their recovery. Following the physical activity engagement, support groups can be conducted to empower participants to adopt healthy and positive lifestyles that push them to avoid relapse. Although further research is needed to determine the therapeutic effects of using exercise in SUD, it is evident that performing physical activity could improve mood and reduce cravings for drugs. Health professionals could also consider utilizing other forms of physical activity requiring little to no specific equipment (eg, yoga or mind-body movement) as an adjunct to SUD treatment.

## Conclusions

Findings of this study indicated that participants perceived positive benefits (ie, pushed forward recovery via running, gained a sense of achievement, and experienced a sense of belonging in the program) to their overall health and recovery while attending the walking/running program. The study evidenced the influence of utilizing exercise as an adjunct treatment on SUD recovery via participants’ perceptions and provided implications for SUD treatment services. More studies of holistic interventions are needed in SUD studies to inform the treatment practices.

## Limitations

Whether participants involved in exercise activities other than the scheduled practices during the program implementation is unknown. Relatedly, participants simultaneously receive services like counseling or medication while participating in the walking and running training program. The data were also limited to participants who continued attending the training; thus, the results could be biased. Several additional limitations include participant’s self-reported assessment, lack of attendance records; the effects of timing of interviews could have on the interview responses, given that the data were collected during the course of the practice or with an immediate follow up after the end of the race. Future research is needed to determine whether behavior change was sustained over time.
